# Performance of CHROMagar ESBL media for the surveillance of extended-spectrum cephalosporin-resistant Enterobacterales (ESCrE) from rectal swabs in Botswana

**DOI:** 10.1099/jmm.0.001770

**Published:** 2023-11-22

**Authors:** Naledi Mannathoko, Ebbing Lautenbach, Mosepele Mosepele, Dimpho Otukile, Kgotlaetsile Sewawa, Laurel Glaser, Leigh Cressman, Laura Cowden, Kevin Alby, Anne Jaskowiak-Barr, Robert Gross, Margaret Mokomane, Giacomo M. Paganotti, Ashley Styczynski, Rachel M. Smith, Evan Snitkin, Tiffany Wan, Warren B. Bilker, Melissa Richard-Greenblatt

**Affiliations:** 1 Department of Biomedical Sciences, University of Botswana, Gaborone, Botswana; 2 Center for Clinical Epidemiology and Biostatistics, Perelman School of Medicine, University of Pennsylvania, Philadelphia, PA, USA; 3 Division of Infectious Diseases, Department of Medicine, Perelman School of Medicine, University of Pennsylvania, Philadelphia, PA, USA; 4 Department of Biostatistics, Epidemiology, and Informatics, Perelman School of Medicine, University of Pennsylvania, Philadelphia, PA, USA; 5 Department of Internal Medicine, University of Botswana, Gaborone, Botswana; 6 Botswana-University of Pennsylvania Partnership (BUP), Gaborone, Botswana; 7 Department of Pathology and Laboratory Medicine, University Pennsylvania, Philadelphia, PA, USA; 8 Department of Pathology and Laboratory Medicine, University of North Carolina, Chapel Hill, NC, USA; 9 Department of Biomedical Sciences, University of Botswana, Gaborone, Botswana; 10 Centers for Disease Control and Prevention (CDC), Atlanta, GA, USA; 11 Department of Microbiology and Immunology, University of Michigan, Ann Arbor, Michigan, USA; 12 Department of Laboratory Medicine and Pathobiology, University of Toronto, ON, Canada; 13 Hospital for Sick Children, Toronto, Ontario, Canada

**Keywords:** antimicrobial resistance, CHROMagar ESBL, extended-spectrum cephalosporin-resistant Enterobacterales, rectal swabs

## Abstract

**Introduction.** Lack of laboratory capacity hampers consistent national antimicrobial resistance (AMR) surveillance. Chromogenic media may provide a practical screening tool for detection of individuals colonized by extended-spectrum beta-lactamase (ESBL)-producing organisms.

**Hypothesis.** CHROMagar ESBL media represent an adequate screening method for the detection of extended-spectrum cephalosporin-resistant *Enterobacterales* (ESCrE), isolated from rectal swabs.

**Aim.** To evaluate the performance of CHROMagar ESBL media to accurately identify ESCrE isolates from rectal swab samples attained from hospitalized and community participants.

**Methodology.** All participants provided informed consent prior to enrolment. Rectal swabs from 2469 hospital and community participants were inoculated onto CHROMagar ESBL. The performance of CHROMagar ESBL to differentiate *Escherichia coli* and *Klebsiella* spp., *Enterobacter* spp. and *Citrobacter* spp. (KEC spp.) as well as select for extended-spectrum cephalosporin resistance were compared to matrix-assisted laser desorption/ionization-time-of-flight MS (MALDI-TOF-MS) and VITEK-2 automated susceptibility testing.

**Results.** CHROMagar ESBL had a positive and negative agreement of 91.2 % (95 % CI, 88.4–93.3) and 86.8 % (95 % CI, 82.0–90.7) for *E. coli* and 88.1 % (95 % CI 83.2–92.1) and 87.6 % (95 % CI 84.7–90.2) for KEC spp. differentiation, respectively, when compared to species ID by MALDI-TOF-MS. When evaluated for phenotypic susceptibilities (VITEK-2), 88.1 % (714/810) of the isolates recovered on the selective agar exhibited resistance to third-generation cephalosporins.

**Conclusion.** The performance characteristics of CHROMagar ESBL media suggest that they may be a viable screening tool for the identification of ESCrE from hospitalized and community participants and could be used to inform infection prevention and control practices in Botswana and potentially other low-and middle-income countries (LMICs). Further studies are required to analyse the costs and the impact on time-to-result of the media in comparison with available laboratory methods for ESCrE surveillance in the country.

## Introduction

The significant rise of multidrug-resistant organisms (MDROs) in hospitals and the community is a public health challenge worldwide, with low- and middle-income countries (LMICs) disproportionately affected [1,3]. Amongst the MDROs, *Enterobacterales* harbouring extended-spectrum beta-lactamases (ESBLs) are leading causes of healthcare-associated infections (HAIs) and are increasingly implicated as pathogens of community-associated infections. The clinical management of infections caused by ESBL-producing bacteria are challenging as these enzymes hydrolyse penicillins, extended-spectrum third-generation cephalosporins and monobactams, leaving carbapenems as one of the few remaining treatment options [4]. In addition, several reports have described the co-carriage of carbapenemases in ESBL-producing strains, further complicating patient management [5,6]. A meta-analysis performed on an adult population demonstrated infections due to ESBL-producing *Enterobacterales* (ESBL-PE) to be significantly associated with a higher odds of mortality compared with non-ESBL-PE infection [odds ratio=1.70, 95 % confidence interval (CI): 1.15–2.49] [7].

To successfully manage ESBL-PE and other pathogens with antimicrobial resistance (AMR), an understanding of the local burden of these MDROs is necessary. Implementing robust AMR surveillance and infection prevention and control measures is fundamental for preventing HAIs. In Botswana, monitoring AMR is a national priority but a disparity exists in the availability of laboratory testing infrastructure (e.g. automated identification systems and resources across the different hospitals) with respective facilities having varying capacities to consistently participate in national AMR surveillance programmes. To support and sustain the nascent AMR surveillance project across the country in the long term, the implementation of practical and accurate identification systems for MDROs is needed. Selective chromogenic media for the identification of MDROs represent a simple, cost-effective solution for addressing AMR surveillance challenges in low-resource contexts. Currently, clinical settings in Botswana do not routinely perform screening to identify patients colonized with MDROs. However, these patients can contribute to nosocomial transmission of MDROs, posing a threat to infection control. A previous evaluation of the selective and differential chromogenic agar media CHROMagar ESBL (CHROMagar) demonstrated superior performance relative to other chromogenic media for the identification of ESBL organisms when compared to matrix-assisted laser desorption ionization-time of flight MS (MALDI-TOF-MS) combined with automated susceptibility instrumentation [8].

## Objectives

To evaluate the performance of CHROMagar ESBL media to accurately identify extended-spectrum cephalosporin-resistant *Enterobacterales* (ESCrE) that were isolated from rectal swab samples collected from participants in hospital and community settings in Botswana.

## Methods

### Specimen collection

Specimens were collected as part of the Antibiotic Resistance in Communities and Hospitals (ARCH) project to evaluate the population-based prevalence of colonization with clinically significant antimicrobial-resistant organisms in Botswana [9,10]. The following study was conducted in Gaborone and two semi-rural surrounding villages (Mochudi and Molepolole) between January and September 2020. Across the three regions, adult participants (≥18 years old) were enrolled at three hospitals and six outpatient clinics. Each enrolled clinic patient was invited to refer three individuals (adults or children) to the study. These community participants were enrolled in a manner identical to that of the clinic patients. One rectal swab was collected from each enrolled participant. Rectal swab samples were collected using COPAN Liquid Amies Elution Swab ESwab (Copan Diagnostics), which consists of a flocked swab in 1 ml Liquid Amies medium. All participants signed an informed consent form prior to enrolling in the study. The study protocol was reviewed and approved by the institutional review boards of the University of Botswana (UBR/RES/IRB/BIO128), Botswana Ministry of Health and Wellness (HPDME 13/18/1) and University of Pennsylvania (832784).

### CHROMagar ESBL medium preparation and isolation of ESCrE

After collection, the rectal swabs were kept at 2–8 °C during transportation and on delivery at the lab. Within 24 h of collection, the swabs were directly inoculated onto CHROMagar ESBL for preliminary identification of ESCrE. The medium was prepared from a powdered base according to the manufacturer’s guidelines at the National Health Laboratory, Gaborone, Botswana, and specimens were also processed on-site. Each newly made batch of plates underwent quality control assessment, using wild-type *Escherichia coli* (ATCC 25922) and the ESBL-producing (SHV-18) *Klebsiella pneumoniae* (ATCC 700603) as negative and positive controls, respectively. Colonies depicting the characteristic morphology for *Escherichia coli* (dark pink to reddish colonies) and *Klebsiella*, *Enterobacter* or *Citrobacter* (KEC) species (metallic blue colonies, with or without a reddish halo) as per the manufacturer’s guidelines, were then sub-cultured onto blood agar plates to obtain pure cultures. Pure isolates were stored in trypticase soy broth-glycerol stocks at −80 °C.

### Confirmatory identification and antimicrobial susceptibilities

Confirmatory isolate identification and phenotypic antimicrobial susceptibility testing (AST) were performed at the Hospital of the University of Pennsylvania Clinical Microbiology Laboratory (Philadelphia, PA, USA). All bacterial stock isolates were shipped on dry ice and stored at −80 °C until further testing. Prior to confirmatory testing, isolates were sub-cultured twice on trypticase soy agar with 5 % sheep blood (Fisher Scientific catalogue no. B21261X). Identification was performed using MALDI-TOF-MS, and the phenotypic AST profile was determined using the VITEK-2 with the AST-GN89 card (bioMérieux). ESCrE were regarded as those isolates demonstrating resistance to ceftriaxone or ceftazidime (third-generation cephalosporins) according to the Clinical Laboratory Standards Institute M100 32^nd^ edition breakpoints.

### Statistical analysis

Frequencies with percentages were generated for categorical variables, such as presumptive identification, species identification and polymicrobial growth in initial rectal swab cultures. Performance characteristics (percentage agreement) were calculated using MedCalc statistical software version 20.218 with MALDI-TOF-MS as the comparator method. Only isolates displaying colours representing *Enterobacterales* (dark pink/red and metallic blue) were included as part of the analysis when evaluating the ability of the CHROMagar to select for ESBLs and differentiate between *E. coli* and KEC spp. For example, false positives were dark pink/red colonies and metallic blue colonies that were identified as organisms other than *E. coli* or KEC spp., respectively. False negatives were *E. coli* isolates displaying metallic blue colonies or KEC spp. that were dark pink/red.

## Results

### Clinical isolate morphology characteristics from rectal samples

In the initial study, 2469 participants were enrolled from the hospital (469/2469; 19.0 %), outpatient clinic (959/2469; 38.8 %) and community (adults: 477/2469; 19.3 % and children 564/2469; 22.8 %) settings. Of the total enrolled participants, 34.6 % (855/2469) had at least one organism growing on the CHROMagar ESBL media. Of the 855 positive cultures, 124 (14.5 %) had more than one presumptive ESCrE organism based on the expected characteristic colony colour (and other morphological differences, i.e. size, hue, texture). The majority of these polymicrobial cultures (118/124; 95.2 %) had two morphologically distinct organisms that were identifiable; however, a small number of cultures (6/124; 4.8 %) produced three discernible isolates with distinct colony morphologies. For this study, 810 isolates that demonstrated a culture colony morphology that was characteristic of presumptive *E. coli* or presumptive KEC species were included in subsequent analyses.

Most of the presumptive ESCrE (543/810; 67.0 %) were a dark pink to red colour and therefore were regarded as *E. coli* based on the manufacturer’s CHROMagar ESBL interpretation. Presumptive KEC represented 31.2 % (253/810) of isolates with the expected characteristic morphology (metallic blue colonies). Fourteen (1.8 %) isolates grew as greenish-blue colonies. This was an atypical morphology and the closest probable presumptive identification was *Pseudomonas* spp., which are expected to yield translucent, cream to pale blue colonies. Although the greenish-blue colony morphology is not included in the manufacturer’s interpretations, the isolates were retained for further testing to determine if they were KEC isolates or possibly *Pseudomonas* spp. Other colony morphologies such as colourless, cream and light brown were observed on a number of primary cultures but were not retained for further testing because they did not exhibit the colony morphology of the targeted *Enterobacterales* species in this study.

### Performance of CHROMagar ESBL in differentiating *E. coli* and KEC isolates in comparison to MALDI-TOF MS

All isolates were then tested with MALDI-TOF-MS for confirmatory species identification (Table 1). Most isolates (783/810; 96.7 %) were identified as either *Escherichia* spp. or KEC species. *Escherichia* was the predominant genus (560/810; 69.1 %) isolated from the CHROMagar ESBL media. Fewer isolates were identified as KEC species (223/810; 27.5 %). Of these, the majority were identified as *K. pneumoniae* (122/223; 54.7 %), followed by *Citrobacter* spp. (78/223; 35.3 %). Nine (64.3 %) of the 14 isolates that had an unexpected greenish-blue colour were identified as KEC spp. by MALDI-TOF-MS and the remaining greenish-blue colonies were *E. coli* (2/14; 14.3 %), *Pseudomonas* spp. (2/14; 14.3 %) and unidentified Gram-negative bacteria (1/14; 7.1 %).

**Table 1.** Comparison of bacterial species identification and resistance to third-generation cephalosporins by CHROMagar ESBL media with MALDI-TOF-MS and VITEK-2

CHROMagar ESBL colour (no., %)	MALDI-TOF-MS identification	No. of isolates	Resistant to ceftriaxone and ceftazidime (no., %)
Pink: 510 (91.1)Blue: 48 (8.6)Green: 2 (0.4)	*Escherichia* spp.	560	551 (98.4)
Pink: 11 (9.0)Blue: 108 (88.5)Green: 3 (2.5)	*Klebsiella* spp.	122	112 (91.8)
Pink: 3 (13.0)Blue: 19 (82.6)Green: 1 (4.3)	*Enterobacter* spp.	23	19 (82.6)
Pink: 15 (19.2)Blue: 58 (74.4)Green: 5 (6.4)	*Citrobacter* spp.	78	17 (21.8)
Pink: 2 (25.0)Blue: 6 (75.0)Green: 0 (0.0)	*Proteus* spp.	8	8 (100.0)
Pink: 0 (0.0)Blue: 6 (100.0)Green: 0 (0.0)	*Kluyvera* spp.	6	0 (0.0)
Pink: 0 (0.0)Blue: 1 (33.3)Green: 2 (66.7)	*Pseudomonas* spp.	3	2 (66.7)
Pink: 0 (0.0)Blue: 1 (100.0)Green: 0 (0.0)	*Aeromonas* spp.	1	1 (100.0)
Pink: 2 (66.7)Blue: 1 (33.3)Green: 0 (0.0)	*Acinetobacter* spp.	3	2 (66.7)
Pink: 0 (0.0)Blue: 3 (75.0)Green: 1 (25.0)	Unidentified, GNB	4	1 (25.0)
Pink: 0 (0.0)Blue: 2 (100.0)Green: 0 (0.0)	Unidentified, GPC	2	1 (50.0)
**Pink: 543** (67.0)**Blue: 253** (31.2)**Green: 14** (1.7)		**810**	**714** (88.1)

KEC, *Klebsiella* species/*Enterobacter* species/*Citrobacter* species*.*

Among the total isolates selected for the study, 13.1 % (106/810) depicted a CHROMagar ESBL morphology that was inconsistent with the MALDI-TOF-MS identification (Table 2). Of the isolates initially regarded as *E. coli* (dark pink/reddish colony morphology), 6.1 % (33/543) were identified as a different species and were mostly identified as *Citrobacter* spp. and *K. pneumoniae* (26/33; 78.8 %). Similarly, of the 267 isolates that initially exhibited the metallic blue colony colour, 27.3 % (73/267) were identified as species that were not KEC spp., the majority being *E. coli* (50/73; 68.5 %). Notably, 3.3 % (27/810) of the isolates were identified by MALDI-TOF-MS as organisms unrelated to *E. coli* or KEC, despite having depicted the expected characteristic *E. coli* or KEC spp. colony colour on the CHROMagar ESBL media (Table 2). For example, other *Enterobacterales* species such as *Proteus* spp. and *Kluyvera* spp. (14/810; 1.7 %) displayed the expected characteristic colony colour of presumptive *E. coli* and presumptive KEC on the media. Additional misidentified organisms by CHROMagar included *Acinetobacter* spp. (3/810; 0.4 %), unidentified Gram-negative bacteria (4/810; 0.5 %) and Gram-positive bacteria (2/810; 0.3 %).

**Table 2.** Summary of misidentified colonies by CHROMagar ESBL media when compared to MALDI-TOF-MS for isolate identification

CHROMagar ESBL colony colour	No. isolated	CHROMagar presumptive identification	Total no. of isolates misidentified (%)	Species misidentified by CHROMagar	No. (%) of species misidentified
Dark pink/red	543	*Escherichia coli*	33 (6.1 %)	*Citrobacter freundii* complex	15 (45.4 %)
				*Klebsiella pneumoniae*	11 (33.3 %)
				*Enterobacter cloacae* complex	3 (9.1 %)
				*Acinetobacter baumannii*	2 (6.1 %)
				*Proteus mirabilis*	2 (6.1 %)
Metallic blue	267	KEC spp.	73 (27.3 %)	*Escherichia coli*	50 (68.5 %)
				*Proteus mirabilis*	6 (8.2 %)
				*Kluyvera ascorbate*	4 (5.5 %)
				Unidentified, GNB	4 (5.5 %)
				*Kluyvera cryocrescens*	2 (2.7 %)
				*Pseudomonas aeruginosa*	2 (2.7 %)
				Unidentified, GPC	2 (2.7 %)
				*Acinetobacter baumannii*	1(1.4 %)
				*Aeromoas caviae*	1 (1.4 %)
				*Pseudomonas mendocina*	1 (1.4 %)

GNB, Gram-negative bacilli; GPC, Gram-positive cocci; KEC, *Klebsiella* species/*Enterobacter* species/*Citrobacter* species*.*

The performance of the CHROMagar ESBL for the differentiation of *E. coli* and KEC spp. was calculated as the percentage agreement based on the dark pink/red and metallic blue colony morphology (Table 3). For dark pink/red colonies, when compared to the identification obtained from MALDI-TOF-MS, CHROMagar ESBL had a positive and negative agreement of 91.2 % [95 % confidence interval (CI), 88.4–93.3] and 86.8 % (95 % CI, 82.0–90.7), respectively. The positive (88.1 %, 95 % CI 83.2–92.1) and negative (87.6 %, 95 % CI 84.7–90.2) agreement for the ESBL CHROMagar for metallic blue colonies compared to the MALDI-TOF-MS identification as KEC spp. was similar to the agar’s performance for dark pink/red colonies.

**Table 3.** Performance of CHROMagar ESBL in differentiating *E. coli* and KEC isolates when using MALDI-TOF-MS as a comparator

	MALDI-TOF-MS isolate identification (no.)		
ESBL CHROMagar colony colour	*E. coli*	KEC spp.	Other
Dark pink/red	510	29	4
Metallic blue	50	194	23

KEC, *Klebsiella* species/*Enterobacter* species/*Citrobacter* species.

### Performance of CHROMagar ESBL for the selection of ESCrE

All 810 presumptive *E. coli* and KEC isolates that had been selected from CHROMagar ESBL cultures were further assessed for resistance to third-generation cephalosporins (ceftriaxone or ceftazidime) with the VITEK-2 system. The majority (714/810; 88.1 %) of the total isolates showed resistance to ceftriaxone and/or ceftazidime (Table 1). However, 11.9 % (96/810) demonstrated susceptibility to both ceftriaxone and ceftazidime, and these were mainly *Citrobacter* spp. (Table S1, available in the online version of this article).

### Impact of polymicrobial cultures on CHROMagar ESBL specificity

Lastly, we observed that the majority of inconsistencies between the CHROMagar ESBL and MALDI-TOF-MS species identification of isolates occurred amongst those that were isolated from polymicrobial cultures (CHROMagar ESBL culture comprising two to three different colonies with distinct morphologies). Generally, MALDI-TOF-MS identified colonies isolated from polymicrobial cultures as the same bacterial species even though some of the isolates had displayed a colony morphology that was inconsistent with the MALDI-TOF-MS result (Table S2). In several instances, isolates from the respective polymicrobial cultures exhibited dissimilar antibiotic susceptibility profiles, suggesting that isolates were possibly different strains of the same bacterial species (Table S3).

## Discussion

In this study we evaluated the performance of CHROMagar ESBL media to differentiate *E. coli* and KEC spp. and to select for ESCrE. The media exhibited good performance for the selection of *E. coli* and KEC spp. demonstrating extended-spectrum cephalosporin resistance when compared to results generated from MALDI-TOF-MS and VITEK-2 for identification and AST, respectively. The findings from this study suggest the utility of CHROMagar ESBL as a screening tool for ESCrE colonization surveillance in hospital and community settings in Botswana.

Over the last decade, there has been a rapid expansion in the range of chromogenic culture media available to clinical laboratories. Often a clear advantage over conventional culture media can be demonstrated as it allows for the selection and differentiation of pathogens, including those that are antibiotic-resistant [11]. Clinical laboratory workflows that rely on conventional non-selective media for screening (e.g. blood agar plates) require further colony identification and susceptibility testing, increasing turnaround times. CHROMagar ESBL provides preliminary results following overnight incubation, whereas non-selective media may require an additional 8–48 h depending on the methodologies available (e.g. MALDI-TOF-MS, automated systems, biochemical, disc diffusion, Etests). Additionally, due to the low complexity and limited infrastructure requirements, CHROMagar ESBL enables surveillance in settings where there is limited to no microbiology capacity available. Earlier detection supports prompt decisions regarding the management of colonized patients in accordance with local infection control policies. In turn, these precautions may limit the nosocomial transmission of ESBLs in hospital settings.

The ability of laboratories to make their own CHROMagar ESBL plates from powder provides flexibility of use in LMICs. Laboratories can prepare plates on demand based on clinical/surveillance needs and not rely on the supply chains in these settings. If there are issues with local refrigeration space and power outages, reducing the quantity of stored plates can also save on resources. While there are potential benefits of CHROMagar EBSL to improve access to ESCrE surveillance and reduce turnaround time for results, cost comparison studies are needed to determine the feasibility of implementation. Although based on environmental sampling, we found the use of CHROMagar to be a cost-effective approach to estimate ESBL bioburden in a neonatal intensive care unit in Botswana (US$269.40 to collect, plate and analyse 50 samples) [12].

CHROMagar ESBL could also be used at the community level for ESBL-PE surveillance to identify sources of ESBL-PE transmission. A growing amount of evidence points to community reservoirs as a significant driver for the emergence and spread of ESBL-PE [13]. ESBL-PE-colonized individuals who were discharged from hospitals have been found to be important sources of ESBL-PE transmission in households [14,15]. Furthermore, with increasing antibiotic use in agriculture, individuals working in animal husbandry have also been found to be a source of ESBL-PE [16,19]. In Botswana, subsistence livestock farming is customary, and recent research data have shown that individuals having contact with swine is an independent risk factor for ESCrE colonization [10]. However, as there are minimal data on other community sources of ESBL-PE in Botswana, CHROMagar ESBL media could be used to monitor the colonization rates in specific populations and frequency of detection in agricultural and environmental samples in future studies and surveillance programmes.

It is noteworthy that 11.9 % of the isolates that grew on the CHROMagar ESBL media were susceptible to both ceftazidime and ceftriaxone. The majority of these isolates were observed on plates containing multiple organisms, including at least one exhibiting resistance to a third-generation cephalosporin. Since ESBL-producing bacteria inactivate β-lactams in the surrounding medium, we propose that the bacteria that were susceptible to ceftazidime or ceftriaxone may satellite around ESBL-PE colonies and this would enable them to grow in the media, leading to the generation of false positives. These findings suggest that CHROMagar ESBL culture specificity may be impacted when there is polymicrobial growth arising from the presence of several bacterial species coexisting in a clinical sample. Furthermore, several isolates that were susceptible to both ceftazidime and ceftriaxone were identified as *Citrobacter* spp. Although the manufacturer indicates CHROMagar ESBL is able to inhibit AmpC producers, such as *Citrobacter freundii* and *Enterobacter* spp. not harbouring an ESBL, it is plausible that these isolates exhibiting susceptibility to third-generation cephalosporins may have been induced to produce an AmpC β-lactamase under the conditions tested.

It is unclear the exact mechanism of how the polymicrobial cultures resulted in some CHROMagar ESBL colony morphologies being inconsistent with MALDI-TOF-MS identification in this study. In many instances, colonies with an inconsistent CHROMagar ESBL morphology were given the same identification by the MALDI-TOF-MS as another organism isolated from the primary plate. It is plausible that incorrect identification resulted from the selection of colonies that were not sufficiently isolated on plates exhibiting overgrowth. These may have been challenging to visually differentiate and identify in the presence of a larger overlapping chromogenic colony. Presumably cross-leaching of enzymes between colonies that are overlapping or within close proximity to one another may have also influenced colony colour. Consequently, CHROMagar ESBL specificity may be improved by successive subculturing of colonies selected from mixed primary cultures to ensure colony purity.

Due to the design of our primary study, limitations existed in assessing the overall performance of CHROMagar ESBL media. First, we intentionally did not select any of the colonies that displayed morphologies inconsistent with *E. coli* and KEC spp., so we cannot assess its performance for other bacterial species. Additionally, percentage agreement for the selection of ESCrE using CHROMagar was unable to be determined as the initial study did not simultaneously inoculate the rectal swabs onto non-selective media. Thus, it is plausible that there were additional unidentified ESCrE not included as part of this analysis due to over-selection by the media. However, these types of studies are challenging as rectal swabs would include an abundance of bacteria and many similar species with possibly different antibiotic susceptibility profiles, which would probably result in missing ESBL-PE. Nonetheless, the results of this study indicated that the performance characteristics of the CHROMagar ESBL media in our setting are similar to previous observations [8,20, 21].

While there have been other studies reporting on the performance characteristics of these chromogenic media, few studies have been done with such a diverse population (in-hospital and community-based participants in Botswana) and many of the published studies have been conducted in high-resource settings. Therefore, this study provides insight as to the performance of CHROMagar ESBL in a low-resource setting in sub-Saharan Africa. These findings will allow hospital epidemiologists and researchers to further assess the benefits of implementing CHROMagar ESBL as a potential screening tool to identify colonized patients and inform implementation of infection prevention and control practices. Prior to implementation, further studies should focus on cost analyses and the impact on the time to result of CHROMagar ESBL in LMICs and compare with current laboratory methods for ESCrE surveillance.

## supplementary material

10.1099/jmm.0.001770 Uncited Table S1.

## Abbreviations

AMR: antimicrobial resistance
CI: confidence interval
ESBL: extended-spectrum beta-lactamase
ESBL-PE: ESBL-producing *Enterobacterales*
ESCrE: extended-spectrum cephalosporin-resistant *Enterobacterales*
HAI: healthcare-associated infection
KEC: *Klebsiella* spp., *Enterobacter* spp. and *Citrobacter* spp.
LMIC: low- and middle-income country
MALDI-TOF: matrix-assisted laser desorption/ionization-time-of-flight
MDRO: multidrug-resistant organism
