# Supplementary material for: Performance of CHROMagar ESBL media for the surveillance of extended-spectrum cephalosporin-resistant Enterobacterales (ESCrE) from rectal swabs in Botswana
Source: J Med Microbiol. 2023 Nov 22;72(11):001770. doi: 10.1099/jmm.0.001770 (PMC11145880; doi:10.1099/jmm.0.001770)
Supplement: Uncited Table S1. [file jmm-72-01770-s001.pdf]

SUPPLEMENTARY TABLES

Table A1. Summary of number of organisms isolated from CHROMagar™ ESBL that were susceptible to both ceftazidime and ceftriaxone when evaluated by automated susceptibility testing using the VITEK-2 system.

MALDI-TOF-MS species identification	Total No. (%)	No. isolates with CHROMagar™ ESBL colony color (%)	
		Metallic blue	Dark pink/red
Citrobacter spp.	61 (63.5)	49	12
Klebsiella pneumoniae	10 (10.4)	9	1
Escherichia coli	9 (9.4)	1	8
Enterobacter cloacae	4 (4.2)	3	1
Kluyvera spp.	6 (6.3)	6	-
GNB	3 (3.1)	3	-
Pseudomonas aeruginosa	1 (1.0)	1	-
Acinetobacter baumannii	1 (1.0)	1	-
GPC	1 (1.0)	1	-
**TOTAL**	**96 (100)**	**74 (77.1)**	**22 (22.9)**

Abbreviations: ESBL, extended-spectrum beta-lactamases; MALDI-TOF-MS, matrix-assisted laser desorption ionization-time of flight- mass spectrometry.

Table A2. Incosistencies between CHROMagar ESBL colony morphology and VITEK-MALDI-TOF identification

Participants	CHROMagar Colony morphology	Prospective ID.	VITEK-MALDI-TOF	No.
Participant-01	Pink colonies	*E. coli*	*Escherichia coli*	
	Dark blue colonies	KEC species	*Escherichia coli*	***1***
Participant-02	Pink colonies	*E. coli*	*Escherichia coli*	
	Dark blue colonies	KEC species	*Escherichia coli*	***2***
Participant-03	Pink colonies	*E. coli*	*Escherichia coli*	
	Dark blue colonies	KEC species	*Escherichia coli*	***3***
Participant-04	Pink colonies	*E. coli*	*Klebsiella pneumoniae*	***4***
	Dark blue colonies	KEC species	*Klebsiella pneumoniae*	
Participant-05	Pink colonies	*E. coli*	*Escherichia coli*	
	Dark blue colonies	KEC species	*Escherichia coli*	***5***
Participant-06	Pink colonies	*E. coli*	*Citrobacter freundii*	***6***
	Dark blue colonies	KEC species	*Citrobacter sedlakii*	
Participant-07	Pink colonies	*E. coli*	*Citrobacter sedlakii*	***7***
	Dark blue colonies	KEC species	*Citrobacter sedlakii*	
Participant-08	Pink colonies	*E. coli*	*Escherichia coli*	
	Dark blue colonies	KEC species	*Escherichia coli*	***8***
Participant-09	Pink colonies	*E. coli*	*Citrobacter sedlakii*	***9***
	Dark blue colonies-type 1	KEC species	*Klebsiella pneumoniae*	
	Dark blue colonies-type 2	KEC species	*Citrobacter sedlakii*	
Participant-10	Pink colonies-type 1	*E. coli*-01	*Escherichia coli*	
	Pink colonies-type 2	*E.coli*-02	*Escherichia coli*	
	Dark blue colonies	KEC species	*Escherichia coli*	***10***
Participant-11	Pink colonies	*E. coli*	*Klebsiella pneumoniae*	***11***
	Dark blue colonies	KEC species	*Klebsiella pneumoniae*	
Participant-12	Pink colonies	*E. coli*	*Klebsiella pneumoniae*	***12***
	Dark blue colonies	KEC species	*Klebsiella pneumoniae*	
Participant-13	Pink colonies	*E. coli*	*Escherichia coli*	
	Dark blue colonies	KEC species	*Escherichia coli*	***13***
Participant-14	Pink colonies	*E. coli*	*Escherichia coli*	
	Dark blue colonies	KEC species	*Escherichia coli*	***14***
Participant-15	Pink colonies	*E. coli*	*Escherichia coli*	
	Dark blue colonies	KEC species	Ox- GNR	***15***
Participant-16	Pink colonies	*E. coli*	*Escherichia coli*	
	Dark blue colonies	KEC species	*Escherichia coli*	***16***
Participant-17	Pink colonies	*E. coli*	*Escherichia coli*	
	Dark blue colonies	KEC species	*Escherichia coli*	***17***
Participant-18	Pink colonies	*E. coli*	*Enterobacter cloacae*	***18***
	Dark blue colonies	KEC species	*Enterobacter cloacae*	
Participant-19	Pink colonies	*E. coli*	*Escherichia coli*	
	Dark blue colonies	KEC species	*Escherichia coli*	***19***

Participant-20	Pink colonies	*E. coli*	*Escherichia coli*	
	Green colonies	KEC.green-01	*Escherichia coli*	***20***
Participant-21	Pink colonies	*E. coli*	*Escherichia coli*	
	Dark blue colonies	KEC species	*Escherichia coli*	***21***
Participant-22	Pink colonies	*E. coli*	*Escherichia coli*	
	Dark blue colonies	KEC species	*Escherichia coli*	***22***
Participant-23	Pink colonies	*E. coli*	*Escherichia coli*	
	Dark blue colonies	KEC species	*Escherichia coli*	***23***
Participant-24	Pink colonies	*E. coli*	*Escherichia coli*	
	Dark blue colonies	KEC species	*Escherichia coli*	***24***
Participant-25	Pink colonies	*E. coli*	*Escherichia coli*	
	Dark blue colonies	KEC species	*Escherichia coli*	***25***
Participant-26	Pink colonies	*E. coli*	*Escherichia coli*	
	Dark blue colonies	KEC species	*Escherichia coli*	***26***
Participant-27	Pink colonies	*E. coli*	*Escherichia coli*	
	Dark blue colonies	KEC species	*Escherichia coli*	***27***
Participant-28	Pink colonies	*E. coli*	*Escherichia coli*	
	Dark blue colonies	KEC species	*Escherichia coli*	***28***
Participant-29	Pink colonies	*E. coli*	*Enterobacter cloacae*	***29***
	Dark blue colonies	KEC species	*Kluyvera cryocrescens*	***30***
	GREEN colonies	KEC.green-02	*Enterobacter cloacae*	
Participant-30	Pink colonies	*E. coli*	*Escherichia coli*	
	Dark blue colonies	KEC species	*Kluyvera cryocrescens*	***31***
Participant-31	Pink colonies	*E. coli*	*Escherichia coli*	
	GREEN colonies	Kleb.green-01	*Escherichia coli*	***32***
Participant-32	Pink colonies	*E. coli*	*Citrobacter sedlakii*	***33***
	Dark blue colonies	KEC species	*Citrobacter sedlakii*	
Participant-33	Pink colonies	*E. coli*	*Klebsiella pneumoniae*	***34***
	Dark blue colonies	KEC species	*Klebsiella pneumoniae*	
Participant-34	Pink colonies	*E. coli*	*Escherichia coli*	
	Dark blue colonies	KEC species	*Escherichia coli*	***35***
Participant-35	Pink colonies	*E. coli*	*Escherichia coli*	
	Dark blue colonies	KEC species	*Escherichia coli*	***36***
Participant-36	Pink colonies	*E. coli*	*Escherichia coli*	***37***
	Dark blue colonies	KEC species	*Escherichia coli*	
Participant-37	Pink colonies	*E. coli*	*Escherichia coli*	***38***
	Dark blue colonies	KEC species	*Escherichia coli*	
Participant-38	Pink colonies	*E. coli*	*Escherichia coli*	
	Dark blue colonies	KEC species	*Escherichia coli*	***39***
Participant-39	Pink colonies	*E. coli*	*Escherichia coli*	
	Dark blue colonies	KEC species	*Escherichia coli*	***40***
Participant-40	Pink colonies	*E. coli*	*Escherichia coli*	
	Dark blue colonies	KEC species	*Proteus mirabilis*	***41***
Participant-41	Pink colonies	*E. coli*	*Escherichia coli*	
	Dark blue colonies	KEC species	*Escherichia coli*	***42***

Participant-42	Pink colonies	*E. coli*	*Citrobacter sedlakii*	***43***
	Dark blue colonies	KEC species	*Citrobacter amalonaticus*	
Participant-43	Dark blue colonies	KEC species	*Aeromonas caviae*	***44***
	Blue colonies	KEC species	*Citrobacter sedlakii/koseri*	
Participant-44	Pink colonies	*E. coli*	*Citrobacter werkmanii*	***45***
	Dark blue colonies	KEC species	*Citrobacter sedlakii*	
Participant-45	Pink colonies	*E. coli*	*Citrobacter sedlakii*	***46***
	Dark blue colonies	KEC species	*Klebsiella pneumoniae*	
Participant-46	Pink colonies	*E. coli*	*Escherichia coli*	
	Dark blue colonies	KEC species	*Escherichia coli*	***47***
Participant-47	Dark blue colonies-large	KEC species	*Escherichia coli*	***48***
	Dark blue colonies-small	KEC species	Gram+ cocci	***49***
Participant-48	Dark blue colonies-large	KEC species	*Klebsiella pneumoniae*	
	Dark blue colonies-small	KEC species	Ox- GNR	***50***
Participant-49	Pink colonies	*E. coli*	*Escherichia coli*	
	Dark blue colonies	KEC species	*Escherichia coli*	***51***
Participant-50	Pink colonies	*E. coli*	*Escherichia coli*	
	Dark blue colonies	KEC species	*Escherichia coli*	***52***
Participant-51	Pink colonies	*E. coli*	*Proteus mirabilis*	***53***
	Dark blue colonies	KEC species	*Proteus mirabilis*	***54***
Participant-52	Pink colonies	*E. coli*	*Enterobacter cloacae*	
	Dark blue colonies	KEC species	*Enterobacter cloacae complex*	***55***
Participant-53	Pink colonies	*E. coli*	*Escherichia coli*	
	Dark blue colonies	KEC species	*Escherichia coli*	***56***
Participant-54	Pink colonies	*E. coli*	*Escherichia coli*	
	Dark blue colonies	KEC species	*Escherichia coli*	***57***
Participant-55	Pink colonies	*E. coli*	*Citrobacter braakii*	***58***
	Dark blue colonies	KEC species	*Citrobacter freundii*	
	Green colonies	KEC.green-02	*Citrobacter braakii*	
Participant-56	Pink colonies	*E. coli*	*Escherichia coli*	
	Dark blue colonies	KEC species	*Escherichia coli*	***59***

Table A3. Discrepancies between CHROMagar ESBL morphology vs. VITEK-MALDI-TOF and ASPs of ‘co-bacteria’ isolates from polymicrobial cultures

**Participant**	**CHROMagar Colony morphology**	**Prospective ID.**	**VITEK-MALDI-TOF**	**Cefz**	**Cefox**	**Ceftaz**	**Ceftri**	**Cefepi**	**Erta**	**Mero**	**Amika**	**Gent**	**Tobr**	**Cipro**	**Levo**	**Tetra**	**Co-tri**	**notes**
**1**	Pink colonies	*E. coli*	*Escherichia coli*	R	S	**R**	**R**	I	S	S	S	S	S	S	S	S	S	
	Dark blue colonies	KEC spp.	*Escherichia coli*	R	I	**S**	**R**	S	I	R	I	S	S	S	S	S	R	Diff
**2**	Pink colonies	*E. coli*	*Escherichia coli*	R	S	**I**	**R**	S	S	S	S	S	S	S	S	R	R	
	Dark blue colonies	KEC spp.	*Escherichia coli*	R	S	**I**	**R**	S	S	S	S	S	S	S	S	R	R	Same
**3**	Pink colonies	*E. coli*	*Escherichia coli*	R	R	**R**	**R**	S	S	S	S	S	S	S	S	R	S	
	Dark blue colonies	KEC spp.	*Escherichia coli*	R	R	**R**	**R**	S	S	S	S	S	S	S	S	R	S	Same
**4**	Pink colonies	*E. coli*	*Klebsiella pneumoniae*	R	S	**S**	**S**	S	S	S	S	R	S	S	S	S	S	Nit-I
	Dark blue colonies	KEC spp.	*Klebsiella pneumoniae*	R	S	**S**	**S**	S	S	S	S	R	S	S	S	S	S	Nit-R
**5**	Pink colonies	*E. coli*	*Escherichia coli*	R	S	**I**	**R**	S	S	S	S	S	S	S	S	R	S	
	Dark blue colonies	KEC spp.	*Escherichia coli*	R	S	**I**	**R**	S	S	S	S	S	S	S	S	R	R	Diff
**6**	Pink colonies	*E. coli*	*Citrobacter freundii*	R	S	**S**	**R**	S	S	S	S	S	S	S	S	R	R	Nit-S
	Dark blue colonies	KEC spp.	*Citrobacter sedlakii*	R	R	**I**	**R**	S	S	S	S	S	S	S	S	S	S	Nit-R
**7**	Pink colonies	*E. coli*	*Citrobacter sedlakii*	R	R	**S**	**S**	S	S	S	S	S	S	S	S	S	S	
	Dark blue colonies	KEC spp.	*Citrobacter sedlakii*	R	R	**S**	**S**	S	S	S	S	S	S	S	S	S	S	Same
**8**	Pink colonies	*E. coli*	*Escherichia coli*	R	S	**S**	**R**	S	S	S	S	S	S	S	S	R	R	
	Dark blue colonies	KEC spp.	*Escherichia coli*	R	S	**I**	**R**	I	S	S	S	S	S	R	I	S	R	Diff
**9**	Pink colonies	*E. coli*	*Citrobacter sedlakii*	R	R	**S**	**S**	S	S	S	S	S	S	S	S	S	S	Nit-S
	Dark blue colonies-type 1	KEC spp.	*Klebsiella pneumoniae*	R	S	**I**	**R**	S	S	S	S	S	S	S	I	S	R	Nit-I
	Dark blue colonies-type 2	KEC spp.	*Citrobacter sedlakii*	R	R	**S**	**S**	S	S	S	S	S	S	S	S	S	S	Nit-S

**10**	Pink colonies- 1	*E. coli*-1	*Escherichia coli*	R	S	**S**	**R**	S	S	S	S	S	S	I	I	R	R	Same
	Pink colonies- 2	*E.coli*-2	*Escherichia coli*	R	S	**S**	**R**	S	S	S	S	S	S	I	I	R	R	Same
	Dark blue colonies	KEC spp.	*Escherichia coli*	R	S	**I**	**R**	R	S	S	S	S	S	S	I	S	R	Diff
**11**	Pink colonies	*E. coli*	*Klebsiella pneumoniae*	R	S	**I**	**R**	I	S	S	S	I	S	S	S	R	R	
	Dark blue colonies	KEC spp.	*Klebsiella pneumoniae*	R	S	**I**	**R**	I	S	S	S	R	S	S	S	R	R	Diff
**12**	Pink colonies	*E. coli*	*Klebsiella pneumoniae*	R	R	**R**	**S**	S	S	S	S	S	S	S	I	S	S	Nit-R
	Dark blue colonies	KEC spp.	*Klebsiella pneumoniae*	R	S	**I**	**R**	S	S	S	S	S	S	S	I	S	S	Nit-S
**13**	Pink colonies	*E. coli*	*Escherichia coli*	R	S	**S**	**R**	S	S	S	S	S	S	R	I	S	R	
	Dark blue colonies	KEC spp.	*Escherichia coli*	R	S	**R**	**R**	R	S	S	S	S	S	S	I	R	R	Diff
**14**	Pink colonies	*E. coli*	*Escherichia coli*	R	S	**R**	**R**	R	S	S	S	S	S	S	I	R	R	Nit-S
	Dark blue colonies	KEC spp.	*Escherichia coli*	R	S	**R**	**R**	R	S	S	S	S	R	S	I	S	R	Nit-I
**15**	Pink colonies	*E. coli*	*Escherichia coli*	R	R	**R**	**R**	S	I	S	S	S	S	S	I	R	R	
	Dark blue colonies	KEC spp.	Ox+ GNR	R	S	**S**	**S**	S	S	S	S	S	S	S	S	S	S	Diff
**16**	Pink colonies	*E. coli*	*Escherichia coli*	R	S	**S**	**R**	S	S	S	S	S	S	S	I	R	S	
	Dark blue colonies	KEC spp.	*Escherichia coli*	R	S	**I**	**R**	I	S	S	S	R	S	S	S	R	R	Diff
**17**	Pink colonies	*E. coli*	*Escherichia coli*	R	S	**I**	**R**	I	S	S	S	S	S	S	S	S	R	
	Dark blue colonies	KEC spp.	*Escherichia coli*	R	S	**I**	**R**	S	S	S	S	S	S	S	S	R	R	Diff
**18**	Pink colonies	*E. coli*	*Enterobacter cloacae*	R	R	**S**	**I**	S	S	S	S	S	S	S	S	S	S	
	Dark blue colonies	KEC spp.	*Enterobacter cloacae*	R	R	**S**	**I**	S	S	S	S	S	S	S	S	S	S	Same
**19**	Pink colonies	*E. coli*	*Escherichia coli*	R	S	**R**	**R**	I	S	S	S	S	S	S	S	R	S	
	Dark blue colonies	KEC spp.	*Escherichia coli*	R	S	**R**	**R**	I	S	S	S	S	S	S	S	R	S	Same
**20**	Pink colonies	*E. coli*	*Escherichia coli*	R	S	**R**	**R**	R	S	S	S	S	S	I	I	R	R	
	Green colonies	KEC. spp	*Escherichia coli*	R	S	**R**	**R**	R	S	S	S	S	S	S	I	R	R	Diff

**21**	Pink colonies	*E. coli*	*Escherichia coli*	R	S	**I**	**R**	S	S	S	S	S	S	I	I	R	R	
	Dark blue colonies	KEC spp.	*Escherichia coli*	R	S	**I**	**R**	I	S	S	S	R	S	I	I	R	R	Diff
**22**	Pink colonies	*E. coli*	*Escherichia coli*	R	S	**I**	**R**	I	S	S	S	S	S	S	S	R	R	
	Dark blue colonies	KEC spp.	*Escherichia coli*	R	S	**I**	**R**	I	S	S	S	S	S	S	S	R	R	Same
**23**	Pink colonies	*E. coli*	*Escherichia coli*	R	S	**S**	**R**	S	S	S	S	S	S	I	I	S	R	
	Dark blue colonies	KEC spp.	*Escherichia coli*	R	S	**S**	**R**	S	S	S	S	S	S	I	I	R	R	Diff
**24**	Pink colonies	*E. coli*	*Escherichia coli*	R	R	**R**	**R**	R	S	S	S	R	R	R	R	R	R	
	Dark blue colonies	KEC spp.	*Escherichia coli*	R	I	**R**	**R**	R	S	S	I	R	R	R	R	R	R	Diff
**25**	Pink colonies	*E. coli*	*Escherichia coli*	R	S	**I**	**R**	I	S	S	S	S	S	S	S	R	R	
	Dark blue colonies	KEC spp.	*Escherichia coli*	R	S	**I**	**R**	S	S	S	S	S	S	S	S	R	R	Diff
**26**	Pink colonies	*E. coli*	*Escherichia coli*	R	S	**I**	**R**	I	S	S	S	S	S	R	R	R	R	
	Dark blue colonies	KEC spp.	*Escherichia coli*	R	S	**R**	**R**	I	S	S	S	S	S	R	R	R	R	Diff
**27**	Pink colonies	*E. coli*	*Escherichia coli*	R	R	**R**	**R**	R	S	S	S	R	R	R	R	R	R	
	Dark blue colonies	KEC spp.	*Escherichia coli*	R	R	**R**	**R**	R	S	S	S	R	R	R	R	R	R	Same
**28**	Pink colonies	*E. coli*	*Escherichia coli*	R	S	**I**	**R**	I	S	S	S	S	S	R	R	R	R	
	Dark blue colonies	KEC spp.	*Escherichia coli*	R	S	**I**	**R**	I	S	S	S	S	S	R	R	R	R	Same
**29**	Pink colonies	*E. coli*	*Enterobacter cloacae*	R	R	**S**	**S**	S	S	S	S	S	S	S	S	S	S	Same
	Dark blue colonies	KEC spp.	*Kluyvera cryocrescens*	R	S	**S**	**S**	S	S	S	S	S	S	S	S	R	R	Diff
	GREEN colonies	KEC spp.	*Enterobacter cloacae*	R	R	**S**	**S**	S	S	S	S	S	S	S	S	S	S	Same
**30**	Pink colonies	*E. coli*	*Escherichia coli*	R	S	**R**	**R**	R	S	S	S	S	S	S	I	R	R	
	Dark blue colonies	KEC spp.	*Kluyvera cryocrescens*	R	S	**S**	**S**	S	S	S	S	S	S	S	S	S	S	Diff
**31**	Pink colonies	*E. coli*	*Escherichia coli*	R	R	**R**	**R**	R	S	S	S	S	S	R	R	R	R	
	GREEN colonies	KEC spp.	*Escherichia coli*	S	S	**S**	**S**	S	S	S	S	S	S	S	S	S	S	Diff
**32**	Pink colonies	*E. coli*	*Citrobacter*	R	R	**S**	**S**	S	S	S	S	S	S	S	S	S	S	Nit-S

			sedlakii															
	Dark blue colonies	KEC spp.	*Citrobacter sedlakii*	R	R	**S**	**S**	S	S	S	S	S	S	S	S	S	S	Nit-I
**33**	Pink colonies	*E. coli*	*Klebsiella pneumoniae*	R	S	**R**	**R**	I	S	S	S	R	I	R	R	S	R	
	Dark blue colonies	KEC spp.	*Klebsiella pneumoniae*	R	S	**R**	**R**	I	S	S	S	R	I	R	I	S	R	Diff
**34**	Pink colonies	*E. coli*	*Escherichia coli*	R	S	**I**	**R**	S	S	S	S	S	S	S	S	R	R	
	Dark blue colonies	KEC spp.	*Escherichia coli*	R	S	**I**	**R**	S	S	S	S	S	S	S	S	R	R	Same
**35**	Pink colonies	*E. coli*	*Escherichia coli*	R	S	**R**	**R**	R	S	S	S	S	S	S	I	R	R	
	Dark blue colonies	KEC spp.	*Escherichia coli*	R	S	**R**	**R**	R	S	S	S	R	S	I	I	I	R	Diff
**36**	Pink colonies	*E. coli*	*Escherichia coli*	R	S	**I**	**R**	I	S	S	S	S	S	R	R	R	S	
	Dark blue colonies	KEC spp.	*Escherichia coli*	R	S	**R**	**R**	S	S	S	S	S	S	R	R	R	S	Diff
**37**	Pink colonies	*E. coli*	*Escherichia coli*	R	R	**R**	**R**	I	S	S	S	S	S	R	I	R	S	
	Dark blue colonies	KEC spp.	*Escherichia coli*	R	S	**R**	**R**	I	S	S	S	S	S	R	I	R	R	Diff
**38**	Pink colonies	*E. coli*	*Escherichia coli*	R	S	**S**	**R**	S	S	S	S	S	S	R	I	S	R	
	Dark blue colonies	KEC spp.	*Escherichia coli*	R	S	**I**	**R**	I	S	S	S	S	S	R	R	R	R	Diff
**39**	Pink colonies	*E. coli*	*Escherichia coli*	R	S	**R**	**R**	I	S	S	S	R	I	R	I	S	R	
	Dark blue colonies	KEC spp.	*Escherichia coli*	R	S	**S**	**R**	I	S	S	S	R	I	R	I	S	R	Diff
**40**	Pink colonies	*E. coli*	*Escherichia coli*	R	S	**R**	**R**	I	S	S	S	S	S	I	S	R	R	
	Dark blue colonies	KEC spp.	*Proteus mirabilis*	R	S	**R**	**R**	R	S	S	S	S	S	S	S	R	S	Diff
**41**	Pink colonies	*E. coli*	*Escherichia coli*	R	I	**R**	**R**	R	S	S	S	S	S	S	S	R	R	
	Dark blue colonies	KEC spp.	*Escherichia coli*	R	S	**R**	**R**	R	S	S	S	R	S	R	R	R	R	Diff
**42**	Pink colonies	*E. coli*	*Citrobacter sedlakii*	R	R	**S**	**S**	S	S	S	S	S	S	S	S	S	S	
	Dark blue colonies	KEC spp.	*Citrobacter amalonaticus*	R	S	**S**	**S**	S	S	S	S	S	S	S	S	S	S	Diff
**43**	Dark blue	KEC spp.	*Aeromonas*	I	S	R	NA	S	NA	S	S	S	NA	S	S	S	S	

	colonies		*caviae*															
	Blue colonies	KEC spp.	*Citrobacter sedlakii/koseri*	R	S	**S**	**S**	S	S	S	S	S	S	S	S	S	S	Diff
**44**	Pink colonies	*E. coli*	*Citrobacter werkmanii*	R	R	**I**	**S**	S	S	S	S	S	S	S	S	S	S	
	Dark blue colonies	KEC spp.	*Citrobacter sedlakii*	R	R	**S**	**S**	S	S	S	S	S	S	S	S	S	S	Diff
**45**	Pink colonies	*E. coli*	*Citrobacter sedlakii*	R	I	**S**	**S**	S	S	S	S	S	S	S	S	S	S	
	Dark blue colonies	KEC spp.	*Klebsiella pneumoniae*	R	S	**S**	**R**	S	S	S	S	S	S	S	S	R	S	Diff
**46**	Pink colonies	*E. coli*	*Escherichia coli*	R	I	**I**	**S**	S	S	S	S	S	S	S	S	R	S	
	Dark blue colonies	KEC spp.	*Escherichia coli*	R	S	**I**	**R**	S	S	S	S	S	S	S	S	R	R	Diff
**47**	Dark blue colonies-large	KEC spp.	*Escherichia coli*	R	S	**R**	**R**	S	S	S	S	S	S	S	S	R	R	
	Dark blue colonies-small	KEC spp.	Gram+ cocci	NA	NA	NA	NA	NA	NA	NA	NA	NA	NA	NA	NA	NA	NA	
**48**	Dark blue colonies-large	KEC spp.	*Klebsiella pneumoniae*	R	S	**R**	**R**	S	S	S	S	S	S	S	S	R	R	
	Dark blue colonies-small	KEC spp.	Ox- GNR	R	S	**S**	**S**	S	S	S	S	S	S	S	S	S	S	Diff
**49**	Pink colonies	*E. coli*	*Escherichia coli*	R	S	**I**	**R**	I	S	S	S	S	S	R	R	R	S	Nit-S
	Dark blue colonies	KEC spp.	*Escherichia coli*	R	S	**I**	**R**	I	S	S	S	S	S	R	R	R	S	Nit-I
**50**	Pink colonies	*E. coli*	*Escherichia coli*	R	S	**I**	**R**	S	S	S	S	S	S	S	S	R	R	
	Dark blue colonies	KEC spp.	*Escherichia coli*	R	S	**I**	**R**	S	S	S	S	S	S	S	S	R	R	Same
**51**	Pink colonies	*E. coli*	*Proteus mirabilis*	R	S	**R**	**R**	I	S	S	S	S	S	S	I	R	R	
	Dark blue colonies	KEC spp.	*Proteus mirabilis*	R	S	**R**	**R**	I	S	S	S	S	S	S	I	R	R	Same
**52**	Pink colonies	*E. coli*	*Enterobacter cloacae*	R	R	**R**	**R**	I	R	S	S	S	S	S	S	R	R	
	Dark blue colonies	KEC spp.	*Enterobacter cloacae complex*	R	R	**R**	**R**	I	I	S	S	S	S	S	S	S	S	Diff
**53**	Pink colonies	*E. coli*	*Escherichia coli*	R	S	**I**	**R**	I	S	S	S	S	S	S	S	R	R	

	Dark blue colonies	KEC spp.	*Escherichia coli*	R	S	**R**	**R**	S	S	S	S	S	S	S	S	R	R	Diff
**54**	Pink colonies	*E. coli*	*Escherichia coli*	R	I	**R**	**R**	R	S	S	S	S	S	S	S	R	R	
	Dark blue colonies	KEC spp.	*Escherichia coli*	R	I	**R**	**R**	R	S	S	S	S	S	S	S	R	R	Same
	Pink colonies	*E. coli*	*Citrobacter braakii*	R	R	**S**	**S**	S	S	S	S	S	S	S	S	R	S	Same
**55**	Dark blue colonies	KEC spp.1	*Citrobacter freundii*	R	R	**S**	**S**	S	S	S	S	S	S	S	S	R	S	Same
	Green colonies	KEC spp.2	*Citrobacter braakii*	R	R	**R**	**R**	I	S	S	S	R	I	R	R	R	R	Diff
**56**	Pink colonies	*E. coli*	*Escherichia coli*	R	S	R	R	R	S	S	S	S	S	I	I	R	R	
	Dark blue colonies	KEC spp.	*Escherichia coli*	R	R	R	R	R	R	S	S	R	S	R	R	R	R	Diff

KEY:ASP: Antibiotic susceptibility profile; Cefz- Cefazolin; Cefox- Cefoxitin; Ceftaz- Ceftazidime; Ceftri- Ceftriaxone; Cefepi- Cefepime; Erta- Ertapenem; Mero- Meropenem; Amika- Amikacin; Gent- Gentamicin; Tobr- Tobramycin; Cipro- Ciprofloxacin; Levo- Levofloxacin; Tetra- Tetracycline; Co-Tri- Trimethoprim/Sulfamethoxazole; Nit-Nitrofurantoin
